# Environmental exposure to persistent organic pollutants measured in breast milk of lactating women from an urban area in central Poland

**DOI:** 10.1007/s11356-020-10767-3

**Published:** 2020-09-18

**Authors:** Peter Grešner, Marek Zieliński, Danuta Ligocka, Kinga Polańska, Wojciech Wąsowicz, Jolanta Gromadzińska

**Affiliations:** 1 Department of Toxicology and Carcinogenesis, Nofer Institute of Occupational Medicine, 8, Sw. Teresy St., 91-348 Lodz, Poland; 2 Department of Biological and Environmental Monitoring, Nofer Institute of Occupational Medicine, Lodz, Poland; 3 Bureau of Quality Assurance, Nofer Institute of Occupational Medicine, Lodz, Poland; 4 Department of Environmental Epidemiology, Nofer Institute of Occupational Medicine, Lodz, Poland

**Keywords:** PCDDs, PCDFs, dl-PCBs, Breast milk, POPs, Environmental exposure

## Abstract

Mothers’ milk is considered a channel by means of which new-borns are exposed to polychlorinated dibenzo-p-dioxins (PCDDs), polychlorinated dibenzofurans (PCDFs), and dioxin-like polychlorinated biphenyls (dl-PCBs), environmental pollutants entering food chain and accumulating in fat-rich tissues. In this study, the concentrations of selected PCDDs, PCDFs, and dl-PCBs (a total of 29 substances) in milk samples of 110 breast-feeding women from an urban area were analyzed using the high-resolution gas chromatography/high-resolution mass spectrometry method. Environmental exposure to these substances was expressed by means of the World Health Organization Toxicity Equivalent (WHO-TEQ_2005_) using the Toxicity Equivalent Factor values from van der Berg et al. (*Toxicol*. *Sci*. 93: 223-241, 2006). Concentrations and WHO-TEQ_2005_ values were then searched for plausible relationships with selected demographic and diet-related factors. The total WHO-TEQ_2005_ toxicity equivalent for all 29 substances was (mean ± SD) 10.57 ± 4.57 pg/g fat, while the WHO-TEQ_2005_ levels of PCDDs/PCDFs and dl-PCBs were 7.90 ± 4.17 pg/g fat and 2.67 ± 1.36 pg/g fat, respectively. The concentration and WHO-TEQ_2005_ toxicity equivalent of dl-PCBs correlated significantly with the mothers’ age (*r*_P_ = 0.3814, *p* < 0.00005; *r*_P_ = 0.2817, *p* < 0.005, respectively). The total WHO-TEQ_2005_ toxicity equivalent for all analyzed substances was found to be positively associated with the frequency of consumption of fish and dairy products (*p* < 0.05 for both associations). These outcomes must, however, be interpreted cautiously due to limited size of the study. The results of this paper may provide a basis for further studies on the exposure to PCDDs, PCDFs, and dl-PCBs, and mechanisms underlying their action.

## Introduction

Polychlorinated dibenzo-p-dioxins (PCDDs) and polychlorinated dibenzofurans (PCDFs) constitute a group of environmental pollutants belonging to the broader group of Persistent Organic Pollutants (POPs). They are produced during many industrial processes, especially in combustion ones and can be found in trace amounts in almost all compartments of the global ecosystem environment. Dioxins are widespread throughout the world and occur in small amounts in the air, soil, water, sediments, and in foods: in dairy products, meat, fish, and crustaceans (Pavan et al. 2006). In 1997, International Agency for Research on Cancer (IARC) classified the 2,3,7,8-TCDD, a substance with the highest Toxicity Equivalent Factor (TEF) TEF = 1 (Van den Berg et al. 2006), as a group 1 carcinogen. Even though the carcinogenic effects of dioxins have extensively been studied in animal models, they have not yet been proven in humans. In animal experiments, dioxins have also been shown to exert teratogenic, allergenic, and genotoxic effects (Shertzer et al. 1998). Cases of skin allergies caused by contact with dioxins have been documented in humans as a chlorine acne.

Dioxin-like polychlorinated biphenyls (dl-PCBs), in contrast to PCDDs and PCDFs, were abundantly produced and used in the past. They are among the most stable organic compounds being relatively resistant to degradation under the influence of physical and chemical factors. Their unique properties (non-flammability, low chemical reactivity, high resistance, low dielectric constant, low acute toxicity) have resulted in their wide usage in the industry, predominantly as excellent dielectrics (insulation materials for electric wires, motors, transformers), heat exchangers, but as well as components of hydraulic fluids, flame retardants, printing inks, and additives for glues and plastics. According to official reports from the beginning of the 21st century, the global production of these compounds reached 1.5 million tons, but this value is assumed to be significantly underestimated (UNEP 2015; Urbaniak 2007; Fielder 2001; Breivik et al. 2007).

A diet is the main source of exposure to dioxins and other POPs. They accumulate in fat-rich tissues, including milk. When accumulated over a long period of time, POPs concentrations in tissues can rise considerably and can be harmful even after a long time from the initial exposure (Kumar et al. 2014).

For a new-born, a mother’s milk constitutes the basic source of all necessary nutrients essential for his/her proper development. On the other hand, it also constitutes an important mean by which all the xenobiotics and toxins accumulated in his/her mother’s body (including POPs) enter a child’s organism. Dioxins are then easily adsorbed in the digestive tract of a new-born—in more than 90% of the total uptake (Abraham et al. 1994). Among factors affecting infant’s exposure to dioxins, a considerable role is played by his/her mother’s place of residence—the environmental dioxin pollution is higher in highly urbanized areas then in rural areas (Kamińska et al. 2014; Schuhmacher et al. 2009; Wittsiepe et al. 2007). Other important factors include, obviously, the mother’s diet and exposure to tobacco smoke (Ulaszewska et al. 2011).

Exposure of a child to dioxins in a phase of his/her rapid growth and development may result in irreversible changes in normal development of his/her nervous system (WHO 2010). Many recent studies have also shown that pre- and postnatal exposure to dioxins may affect the development and functioning of other organs and systems, including endocrine glands leading potentially to disruption of the child’s endocrine system (Stølevik et al. 2013; Legler et al. 2011).

In this study, we aimed to determine the environmental exposure of women and their children to PCDDs, PCDFs, and dl-PCBs by measuring concentration of these substances in a lactating mother’s milk. The study also attempted to find statistically significant relationships between concentration of these compounds and selected demographic and diet-related factors (fat-rich diet components, women's age, BMI, the milk fat content).

## Materials and methods

### Study group and sampling

The hereby described study is based on the data from the Polish Mother and Child Cohort study (REPRO_PL), which has been thoroughly described elsewhere (Polańska et al. 2009, 2016). The research material was collected in cooperation with hospitals and obstetric and gynecological clinics within the Lodz district, central Poland, during the REPPRO_PL recruitment period (2007–2011). Women with healthy single pregnancy were recruited for examinations from 8 different regions of Poland. Out of the total of 1266 women with all required data on pregnancy and the new-born recruited within REPRO_PL, only those residing within the urban area of the city of Lodz for at least 5 years with sufficient excess of milk (enough milk for both the child and analysis) were eligible for this study. Ultimately, a total of 110 women aged 16 to 38 years constituted the basis of this study, comprising some 8.7% of subjects enrolled in the REPRO_PL with available data.

Following 3 to 8 weeks after delivery, the milk was collected from all nursing mothers enrolled in the study in an amount of about 100 ml into glass containers during home visits. After delivery to the laboratory, the milk was stored at −20 °C until analysis. Basic characteristics of pregnant women participating in the study, including age, maternal weight before pregnancy, BMI, and milk fat content, were collected from all women meeting the inclusion criteria and are presented in Table 1. Additional information on the frequency of consumption of selected groups of fat-rich food products were also collected from each participant (Table 2).

**Table 1:** Characteristics of pregnant women participating in the study (*N* = 110)

	Mean	SD	Min	Max
Age (years)	27.8	3.7	16.0	38.0
Gestational age (weeks)	39.3	1.2	37.0	42.0
Height (cm)	166	6	155	183
Weight before pregnancy (kg)	60.1	9.8	45.0	100.0
BMI	22.0	3.6	17.0	40.1
Birth weight (g)	3451	472	2500	4600
Milk fat content (g/l)	2.0	0.7	0.6	3.9

**Table 2:** Characteristics of pregnant women participating in the study—the frequency of consumption of selected groups of fat-rich diet components

	Frequency of consumption					
Diet component	Never	Less than once a week	Once a week	Twice a week	More than twice a week	Every day
Fish	8	54	36	10	2	0
Meat	1	1	2	10	47	49
Milk	13	18	8	6	30	35
Dairy products	6	1	4	10	43	46
Eggs	7	23	32	30	18	0

The study was performed under the guidelines of the Helsinki Declaration for human research and was granted an approval issued by the Local Bioethics Committee of the Nofer Institute of Occupational Medicine (No. 7/2007). Written and informed consent for participation in REPRO_PL as well as in this study was obtained from each participant. All data were collected in accordance with the GDPR provision on personal data protection.

### PCDDs/PCDFs and dl-PCBs extractions, clean-up, and analysis

Sample preparation and determination was carried out in accordance with the recommendations of PN-EN 1948-2:2006, PN-EN 1948-3:2006, and U.S. EPA Method 1668, Revision A: Chlorinated Biphenyl Congeners in Water, Soil, Sediment, and Tissue by HRGC/HRMS. US EPA 1999. In accordance with the above-mentioned standards and the Regulation of the Minister of Health for the determination of seven 2,3,7,8-substituted PCDDs, ten 2,3,7,8-substituted PCDFs, and twelve dl-PCBs, it is mandatory to use the high-resolution gas chromatography/high-resolution mass spectrometry (HRGC/HRMS). Determination of PCDD/PCDF and dl-PCB (as well as fat content) was performed according to the procedure of Kamińska et al. (2014). The developed procedure was accredited by the Polish Centre for Accreditation (PCA, certificate No. AB 215).

Concentrations of PCDDs, PCDFs, and dl-PCBs in analyzed samples were calculated using the QuanLynx/TargetLynx program. Toxicity of samples was then expressed by means of the World Health Organization Toxicity Equivalent (WHO-TEQ_2005_), which is the sum of the concentration × TEF products for considered compounds in the sample. The methodology is thoroughly described in the study by Kamińska et al. (2014).

### Statistical analysis

Concentrations and WHO-TEQ_2005_ toxicity equivalents of each compound from the PCDDs, PCDFs, and dl-PCBs groups were both characterized by standard descriptive statistics including mean, standard deviation (SD), median, the upper (UQ) and lower (LQ) quartiles, and minimum (MIN) and maximum (MAX) values. Simple correlations between the parameters describing exposure to analyzed substances on one side and age, BMI, and milk fat content on the other one were characterized by Pearson’s correlation coefficient (*r*_P_). Simple between-group comparisons were investigated using the Kruskal-Wallis *H* test or the Mann-Whitney *U* test. Statistical significance was inferred for *p* < 0.05.

All statistical analyses were conducted in Statistica 10 package (StatSoft, Tulsa, OK, USA).

## Results

### Concentrations of PCDDs, PCDFs, and dl-PCBs in lactating mothers’ milk

Seven PCDDs, 10 PCDFs, and 12 dl-PCBs were determined in 110 milk samples collected from the nursing women residing in Lodz, Poland, for a minimum period of 5 years. Out of the hereby analyzed substances, the lowest detection rate was observed for 1,2,3,4,7,8-HxCDD, which we were able to detect in 74.5% of subjects, while OCDD was the substance with the highest detection rate of 98.2% of subjects. Detection rates for all other substances were within these boundaries. Respective limits of quantification (LOQ) as well as detection rates for all substances analyzed in this study are provided in Table 3.

**Table 3:** Summary of concentrations and WHO-TEQ_2005_ toxicity equivalents for PCDDs, PCDFs, and dl-PCBs congeners measured in the milk of the lactating women (*N* = 110) living in Lodz, Poland

Chemical substance	LOQ	> LOQ (%)	Mean	SD	Min	LQ	Median	UQ	Max	IQR
2378-TCDD	0.1	84.5	0.90	0.73	0.1	0.27	0.81	1.36	2.69	1.09
12378-PeCDD	0.5	87.3	2.38	1.92	0.5	0.90	2.19	3.49	9.09	2.58
123478-HxCDD	0.5	74.5	1.64	3.22	0.5	0.02	0.78	2.12	29.72	2.10
123678-HxCDD	0.5	90.9	5.45	3.04	0.5	3.45	5.54	7.32	14.81	3.87
123789-HxCDD	0.5	84.5	3.11	13.18	0.5	0.45	1.50	2.79	138.54	2.34
1234678-HpCDD	0.5	94.5	7.69	5.07	0.5	4.59	6.89	9.64	40.05	5.05
OCDD	1.0	98.2	37.66	25.36	1.0	23.49	33.09	48.47	218.86	24.98
*Sum of PCDDs (pg/g fat)*			*58.82*	*33.11*	*1.84*	*39.2*	*52.87*	*69.67*	*261.72*	*30.47*
2378-TCDF	0.1	94.5	0.96	1.07	0.1	0.41	0.72	1.20	6.87	0.79
12378-PeCDF	0.5	77.3	1.65	1.98	0.5	0.04	1.17	2.31	9.35	2.27
23478-PeCDF	0.5	96.4	7.54	3.73	0.5	5.37	7.82	9.96	17.77	4.59
123478-HxCDF	0.5	94.5	3.59	2.75	0.5	1.82	3.15	4.71	14.42	2.89
123678-HxCDF	0.5	90.9	2.36	1.96	0.5	1.01	1.89	2.97	9.73	1.96
234678-HxCDF	0.5	88.2	3.08	3.28	0.5	0.83	1.97	4.50	19.04	3.68
123789-HxCDF	0.5	80.0	1.91	3.85	0.5	0.18	0.89	2.59	36.71	2.41
1234678-HpCDF	0.5	92.7	2.66	2.45	0.5	1.08	1.97	3.82	11.45	2.74
1234789-HpCDF	0.5	80.9	1.54	1.97	0.5	0.28	1.01	1.79	10.43	1.51
OCDF	1.0	83.6	4.79	6.03	1.0	0.28	3.28	6..59	29.49	6.31
*Sum of PCDFs (pg/g fat)*			*30.10*	*20.26*	*5.10*	*14.52*	*26.56*	*40.68*	*110.12*	*26.16*
*Sum of PCDFs/PCDDs (pg/g fat)*			*88.93*	*44.8*	*26.79*	*59.14*	*81.07*	*105.3*	*318.47*	*46.16*
*WHO-TEQ of PCDDs/ PCDFs (pg TEQ/g fat)*			*7.90*	*4.17*	*0.52*	*4.82*	*7.42*	*10.41*	*22.81*	*5.59*
PCB-81	0.02	85.5	7.12	12.18	0.02	0.82	3.24	8.28	87.10	7.46
PCB-77	0.02	87.3	6.73	9.57	0.02	1.94	4.33	10.32	86.45	8.38
PCB-123	0.02	90.9	105.22	150.97	0.02	39.39	70.25	132.68	1398.99	93.29
PCB-118	0.12	90.9	3326.21	1804.87	0.12	2484.32	3142.06	4107.66	9569.93	1623.34
PCB-114	0.02	90.0	139.89	82.36	0.02	89.06	140.00	186.50	398.58	97.44
PCB-105	0.02	83.6	617.69	465.37	0.02	394.04	557.30	806.14	2976.09	412.1
PCB-126	0.02	85.5	21.04	12.31	0.02	15.16	20.64	28.27	49.54	13.11
PCB-167	0.02	90.9	427.42	246.74	0.02	291.31	413.72	540.43	1481.54	249.11
PCB-156	0.02	89.1	1223.19	947.58	0.02	777.42	1026.05	1482.58	7164.4	705.16
PCB-157	0.02	90.9	273.53	160.8	0.02	178.22	248.91	361.20	781.70	182.98
PCB-169	0.02	95.5	13.36	12.87	0.02	7.48	10.55	15.41	110.14	7.93
PCB-189	0.02	92.7	144.63	104.87	0.02	83.81	128.39	170.04	701.89	86.23
*Sum of non-orto dl-PCBs (pg/g fat)*			*47.92*	*31.27*	*0.06*	*30.92*	*42.21*	*58.47*	*237.07*	*27.55*
*Sum of mono-orto dl-PCBs (pg/g fat)*			*6143.99*	*3405.45*	*0.26*	*4198.27*	*5808.22*	*7661.49*	*21971.59*	*3463.21*
*Sum of dl-PCBs (pg/g fat)*			*6191.92*	*3413.63*	*18.11*	*4233.4*	*5856.98*	*7723.13*	*22052.71*	*3489.73*
*WHO-TEQ of dl-PCBs (pg TEQ/g fat)*			*2.67*	*1.36*	*0.02*	*1.97*	*2.59*	*3.45*	*6.00*	*1.48*
*WHO-TEQ of PCDDs/PCDFs/dl-PCBs (pg TEQ/g fat)*			*10.57*	*4.57*	*1.59*	*7.32*	*9.63*	*13.32*	*25.17*	*6.01*

Data for the limits of quantification (LOQ) and measured concentrations are provided as pg/g fat, while for the WHO-TEQ_2005_ as pg TEQ/g fat. Italic values refer to sums of PCDD, PCDF, and dl-PCB congeners, and WHO-TEQ_2005_ values calculated for selected POP groups. *SD*, standard deviation; *Min*, minimum; *LD*, lower quartile; *UD*, upper quartile; *Max*, maximum; *IQR*, interquartile range

The mean concentrations of the sum of 7 PCDDs, the sum of 10 PCDFs, and the sum of 12 dl-PCBs were 58.82 ± 33.11 pg/g fat, 30.10 ± 20.26 pg/g fat, and 6191.92 ± 3413.63 pg/g fat, respectively, with corresponding maximum observed values 261.72 pg/g fat, 110.12 pg/g fat, and 22052.71 pg/g fat, respectively. The total WHO-TEQ_2005_ toxicity equivalent (for all substances analyzed in the study) of tested samples ranged from 1.59 to 25.17 pg TEQ/g fat with mean value of 10.57 ± 4.57 pg TEQ/g fat. WHO-TEQ_2005_ for dioxins and furans ranged from 0.52 to 22.81 pg TEQ/g fat, while for dl-PCB from 0.02 to 6.00 pg TEQ/g fat, with mean values of 7.90 ± 4.17 and 2.67 ± 1.36 pg TEQ/g fat, respectively.

In the case of PCDDs and PCDFs, OCDD was found to present the highest mean concentration (37.66 ± 25.36 pg/g fat) representing some 42.3% of all the compounds in this group, followed by 1,2,3,4,6,7,8-HpCDD (7.69 ± 5.07 pg/g fat) and 2,3,4,7,8-PeCDF (7.54 ± 3.73 pg/g fat), constituting 8.7% and 8.5% of the whole amount of compounds in this group, respectively. Compounds with 4 substituted chlorines—TCDF and TCDD, the most toxic one in this group (TEF = 1)—had the smallest mass share in the group of PCDDs/PCDFs compounds: 1.1% and 1.0%, respectively. Furthermore, PeCDD, the second most toxic compound (TEF = 1), constituted on average some 2.7% of compounds in this group. Considering dl-PCBs, PCB-118, PCB-156, and PCB-105 were the three most abundant ones with mean concentrations of 3326.21 ± 1804.87 pg/g fat, 1223.19 ± 947.58 pg/g fat, and 617.69 ± 465.37 pg/g fat, respectively. Among the most toxic PCBs (non-ortho substituted), PCB-126 (21.04 ± 12.31 pg/g fat) and PCB-169 (13.36 ± 12.87 pg/g fat) were the two most abundant. Detailed results are presented in Table 3.

### Relationships between concentrations of PCDDs, PCDFs, and dl-PCBs in lactating mothers’ milk and selected demographic and diet-related factors

Summary of Pearson’s rank correlation analysis between the parameters describing exposure to PCDDs, PCDFs, and dl-PCBs (sum of concentrations, WHO-TEQ_2005_ toxicity equivalents) and women’s age, BMI, and milk fat content is shown in Table 4. Statistically significant correlations were found only for dl-PCBs, in the case of which the total dl-PCB concentration was positively (*r*_P_ = 0.3814, *p* < 0.00005) and negatively (*r*_P_ = − 0.1993, *p* < 0.05) correlated with the women’s age and the milk fat content, respectively, while the corresponding WHO-TEQ_2005_ toxicity equivalent correlated positively with women’s age only (*r*_P_ = 0.2817, *p* < 0.005). Statistically significant relationships between the mothers’ age and parameters related to exposure to dl-PCBs were apparent also following dichotomization of the study group based on median age (28 years): in the subgroup of “younger” mothers (i.e., those with age below the median age), both the summary concentration (below median vs. above median: 4788.5 [3403.8–6564.6] pg/g fat vs. 6907.6 [4772.2–8819.6], *p* < 0.001) and the corresponding WHO-TEQ_2005_ toxicity equivalent (below median vs. above median: 2.35 [1.69–3.06] pg/g fat vs. 2.94 [2.18–3.77], *p* < 0.05) of dl-PCBs were statistically significantly lower when compared with “older” mothers (i.e., those with age above median value). No other statistically significant correlations or differences were found.

**Table 4:** Analysis of correlations between parameters of exposure to PCDDs, PCDFs, and dl-PCBs and women’s age, BMI, and milk fat content

	Age	BMI	Milk fat content
Sum of PCDDs/PCDFs	0.1200	0.0750	− 0.1602
WHO-TEQ of PCDDs/PCDFs	0.0491	0.0488	− 0.0447
Sum of dl-PCBs	0.3814 ^a^	0.0824	− 0.1993 ^c^
WHO-TEQ of dl-PCBs	0.2817 ^b^	0.0666	− 0.0994
Total WHO-TEQ (PCDDs/PCDFs/dl-PCBs)	0.1005	0.0666	− 0.0423

Presented are the values of the Pearson correlation coefficients for tested correlations between the parameters describing exposure and women’s age, BMI, and the milk fat content

^a^*p* < 0.00005

^b^*p* < 0.005

^c^*p* < 0.05

**Fig. 1:**
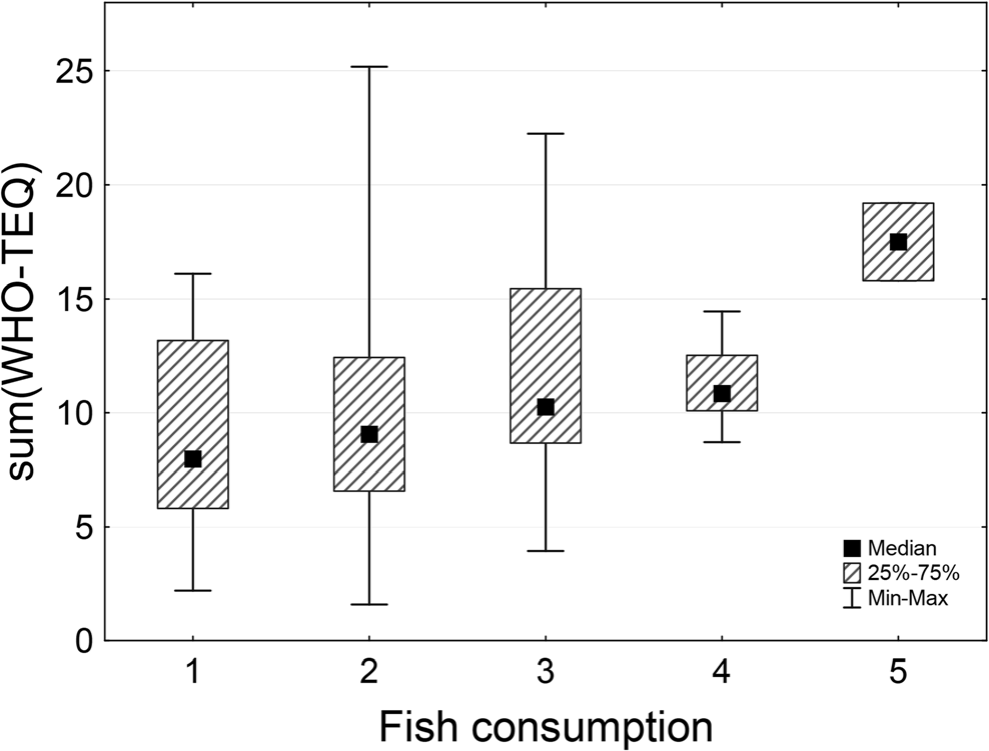
The relationship between the total WHO-TEQ_2005_ toxicity equivalent and the frequency of fish consumption. Shown are the median levels (black squares) together with respective interquartile ranges (shaded rectangles) and min-max ranges (whiskers) of total WHO-TEQ_2005_ toxicity equivalents in individual groups created based on different frequency of fish consumption (1 – never, 2 – less than once a week, 3 – once a week, 4 – twice a week, 5 – more than twice a week). Respective values are provided in Table 5A

Plausible associations between the parameters of exposure and the frequency of consumption of various diet components were also analyzed. The total WHO-TEQ_2005_ toxicity equivalent for all the 29 substances analyzed in the study was considered and as shown in Table 5A, it was found to be significantly associated with the frequency of consumption of fish and dairy products (*p* < 0.05 for both). While the post hoc analysis failed to show any statistically significant differences among individual groups based on the frequency of either fish or dairy products consumption (*data not shown*), the median levels of total WHO-TEQ_2005_ toxicity presented clear progressive increase with increasing fish consumption frequency (Fig. 1). No such clear trend was, however, observed in the case of association between the total WHO-TEQ_2005_ total toxicity equivalent and the frequency of consumption of dairy products (see Table 5A).

**Table 5:** Associations between total WHO-TEQ_2005_ toxicity equivalent and WHO-TEQ_2005_ toxicity equivalents of PCDDs/PCDFs and dl-PCBs with selected diet components

		Frequency of consumption					
		Never	Less than once a week	Once a week	Twice a week	More than twice a week	Every day
A		*WHO-TEQ of PCDDs/PCDFs/dl-PCBs (pg TEQ/g fat)*					
	Fish ^a^	8.0 [5.8−13.2]	9.1 [6.6−12.4]	10.2 [8.7−15.5]	10.8 [10.1−12.5]	17.5 [15.8−19.2]	-
	Meat	15.3 [15.3−15.3]	9.4 [6.3−12.5]	8.8 [6.1−12.8]	11.4 [7.4−15.8]	9.5 [7.3−12.5]	10.2 [7.3−14.1]
	Milk	8.7 [7.2−9.3]	12.5 [9.3−15.6]	11.9 [10.1−19.8]	9.7 [7.9−11.2]	8.8 [7.1−12.0]	9.7 [5.9−12.9]
	Dairy products ^a^	7.1 [5.6−8.7]	16.9 [16.9−16.9]	14.9 [9.5−19.3]	11.3 [7.3−15.6]	9.6 [7.1−12.5]	10.0 [8.8−12.7]
	Eggs	8.3 [7.2−15.9]	11.3 [7.3−16.0]	9.6 [7.9−12.0]	10.2 [7.0−15.0]	9.3 [6.6−12.7]	-
B		*WHO-TEQ of PCDDs/PCDFs (pg TEQ/g fat)*					
	Fish ^b^	5.6 [4.6−10.2]	6.2 [4.0−8.7]	8.2 [5.4−11.0]	8.5 [6.6−9.5]	12.9 [10.6−15.1]	-
	Dairy products ^a^	4.5 [3.4−5.4]	12.4 [12.4−12.4]	12.9 [8.2−14.7]	9.8 [5.8−10.4]	6.5 [4.2−9.2]	7.7 [5.6−10.9]
C		*WHO-TEQ of dl-PCBs (pg TEQ/g fat)*					
	Fish	2.0 [0.7−3.2]	2.5 [1.8−3.4]	2.5 [2.1−3.4]	3.2 [1.6−3.8]	4.6 [4.1−5.2]	-
	Dairy products	2.2 [1.7−3.1]	4.5 [4.5−4.5]	2.7 [1.3−4.7]	2.9 [0.2−3.4]	2.5 [2.1−3.5]	2.6 [2.0−3.2]

Presented are the respective median values (with interquartile ranges in square brackets) of (A) total (B) PCDDs/PCDFs and (C) dl-PCBs WHO-TEQ_2005_ toxicity equivalents in individual groups obtained based on frequency of consumption of selected diet components. Associations between WHO-TEQ_2005_ toxicity equivalents and the frequency of consumption of diet components were tested for significance using the Kruskal-Wallis *H* test, and post hoc analysis of plausible between-group differences was performed using the standard *z*-test

^a^*p* < 0.05

^b^*p* = 0.0648

Due to these interesting outcomes, associations between the fish and dairy products consumption frequency and the WHO-TEQ_2005_ toxicity equivalent of PCDDs/PCDFs and dl-PCBs separately were also analyzed. WHO-TEQ_2005_ of PCDDs/PCDFs was found to be significantly associated with the frequency of dairy products consumption (*p* < 0.05), while its association with the frequency of fish consumption remained close to the edge of statistical significance (*p* = 0.0648). The trend patterns for WHO-TEQ_2005_ of PCDDs/PCDFs in terms of its associations with consumption of fish or dairy products consumption resembled those observed in the case of total WHO-TEQ_2005_ toxicity equivalents (Table 5B). WHO-TEQ_2005_ of dl-PCBs did not show any statistically significant associations with either fish or dairy products consumption frequency (Table 5C).

## Discussion

PCDDs, PCDFs, and dl-PCBs are compounds which, due to their durability and accumulation, are present in various compartments of the environment, from water through sediments or soil to air. From all these compartments, they enter the food directly or indirectly. Food is then considered the main source of intake of dioxins in humans and it is estimated that it constitutes some 90% of human’s overall dioxin intake.

Numerous xenobiotics such as pesticides, plasticizers, or other chemicals intentionally produced and used in the industry or agriculture can be controlled—i.e., their production can be reduced or discontinued, or they can be withdrawn from the market. However, environmental pollution can also be caused by unintentionally introduced factors—as a part of improper or ineffective utilization, uncontrolled combustion, especially in home furnaces, but also during fires, especially extensive and uncontrolled forest fires. PCDDs and PCDFs are two examples of such pollutants. Toxicity, bioaccumulation, and their biomagnification in water and land food chains make these compounds a real long-term threat to humans and animals. Such persistent environmental pollutants can be determined by modern analytical techniques at femtogram level and in virtually any environmental matrix. In subsequent stages of food chain, dioxins accumulate in animal fat and adipose tissue; therefore, the amount of human intake depends strictly on a diet used and the preferred food products.

Significant levels of these organic contaminants are accumulated in fat-rich tissues, including breast milk. Breast-feeding milk contains femto- or picogram amounts of PCDDs, PCDFs, and dl-PCBs per gram of fat. For the first few months of a child’s life, a mother’s milk is its only food. To determine the uptake of the above-mentioned compounds and to assess the effects of such an exposure in infants, proper development of analytical techniques is extremely important. In such studies, the time elapsed between delivery and milk sample collection is crucial, as the milk fat content changes in the subsequent weeks of lactation (Florea 2014). On the other hand, the amount of milk drunk by a growing child rises.

In the hereby described study, the concentrations of 10 PCDFs, 7 PCDDs, and 12 dl-PCBs (including 4 non-ortho dl-PCBs and 8 mono-ortho dl-PCBs) were determined in a lactating mother’s milk samples. The average total WHO-TEQ_2005_ toxicity equivalent (for all the 29 compounds) was 10.57 ± 4.57 pg WHO-TEQ/g fat, while for PCDDs and PCDFs, it was 7.90 ± 4.17 pg WHO-TEQ/g fat. The toxicity equivalent for dl-PCBs alone was only 2.67 ± 1.36 pg WHO-TEQ/g fat, although dl-PCB concentrations were significantly higher than those determined for dioxins and furans. This is due to the lower toxicity of dl-PCBs compared with the one of PCDDs or PCDFs, and especially to the most dangerous of them—2,3,7,8-TCDD and 1,2,3,7,8-PeCDD, for which the value of TEF is equal to 1. Very similar results were obtained in a work by Lu et al. (2015). They have determined the levels of dioxins, furans, and biphenyls in the milk of lactating women living in urban areas in China. The average WHO-TEQ_2005_ in their study was 8.3 pg WHO-TEQ/g fat, which is slightly lower than the value obtained by us, and the WHO-TEQ_2005_ toxicity equivalents for PCDDs/PCDFs and dl-PCBs individually were 2.9 and 5.4 pg WHO-TEQ/g fat, respectively. The authors themselves note that the toxicity equivalents they obtained in their study are lower compared with those reported from other countries, but point, at the same time, to higher concentrations of POPs (especially dl-PCBs) in milk taken from women living in urban areas. In our work, this relationship was also noticed (*data not shown*). The mean concentrations of the most toxic among dioxins (TCDD and PeCDD) were 0.90 ± 0.73 and 2.38 ± 1.92 pg/g fat, respectively, while the mean concentration of the most commonly occurring dioxin—OCDD—was 37.66 ± 25.36 pg/g fat. In the case of dl-PCBs, PCB-118 congener turned out to be the most common compound. This congener is a compound most frequently found in both biological (milk, blood) and environmental (sediments, ashes, or water) matrices (Zieliński et al. 2014, Urbaniak et al. 2008, 2014). Its mean concentration was 3326 ± 1804.87 pg/g fat, while the maximum determined value was as much as 9570 pg/g fat. Similarly, in the case of the OCDD congener, it has also been the most common one among dioxins and significantly affects the total amount of all PCDDs in the sample (Zieliński et al. 2014; Urbaniak et al. 2012, 2014). Nevertheless, its contribution to total toxicity of the entire sample is insignificant. The results obtained by Lu et al. (2015) were similar. OCDD was also predominant among the dioxins, while PCB-118, with its mean concentration of approx. 1896 pg/g fat, definitely exceeded concentrations of other dl-PCBs.

In a study by Manh et al. (2015), reporting the concentrations of dioxins in milk of nursing mothers living in dioxin-contaminated cities in Vietnam, the determined WHO-TEQ_2005_ toxicity equivalent was found to be 9.3 pg WHO-TEQ/g fat. It is also interesting that 2,3,7,8-TCDD, the most dangerous dioxin, constituted over 23% of all measured congeners, while other compounds had a significantly smaller mass share. It is completely different from our findings in the case of which the pollution came only from urban development and traffic, and the mass share of TCDD constituted only some 1%. In the cities distant from places contaminated with dioxins, the share of tetrachlorodibenzo-*p*-dioxin is smaller, while higher concentrations of other compounds, also from the PCDFs group, show up. However, OCDD still remains a congener with the highest concentrations—irrespective of whether measured in cities in dioxin-polluted areas or in the cities away far from such. In Europe, high concentrations of dioxins and biphenyls in milk of lactating women have been found in France (Focant et al. 2013). Although concentrations of the most dangerous dioxins—TCDD and PeCDD—were only 0.80 pg/g fat and 1.62 pg/g fat, respectively, which is slightly lower compared with our study, the total WHO-TEQ_2005_ equivalent was as high as 17.81 pg WHO-TEQ/g fat (compared with 10.57 pg/g fat in our present study). Respective value for dl-PCBs was 7.69 pg WHO-TEQ/g fat, compared with 2.67 pg/g fat in our study. These values were higher than those presented by us mainly due to high concentrations of polychlorinated biphenyls (the highest value for PCB-118) but above all, mainly by high HxCDD values, which were at considerably lower level in our study. Besides that, our data are comparable with those from other places throughout Europe. Comparing the range of PCDDs/PCDFs and PCBs levels in milk determined in the WHO studies between 2006 and 2009, the levels found among the Polish mothers’ milk and reported here in our study surely fit into rather medium and lower part of such range. Out of the cited studies, the highest concentrations were definitely determined in milk samples from Moldova, the Czech Republic, and Luxembourg, where dioxins were found to be at the levels exceeding 20 pg WHO-TEQ/g fat (ranging from 16 to 47 pg WHO-TEQ/g fat). In Belgium and Slovakia, the values were above 15 pg WHO-TEQ/g fat (ranging from 16 to 52 pg WHO-TEQ/g fat). The lowest levels of WHO-TEQ_2005_ were obtained in Fiji and Ghana—below 6 pg WHO-TEQ/g fat (Adu-Kumi et al. 2010). Slightly lower concentrations than those presented in our study were found in samples collected from lactating women in Cyprus (an average of 7.0 pg WHO-TEQ/g fat) and South Korea (an average of 6.5 pg WHO-TEQ/g fat; Adu-Kumi et al. 2010). A large pool of samples (*n* = 300) was analyzed by Canadian researchers (Ryan and Rawn 2014), who reported levels comparable with ours (8.3 pg WHO-TEQ/g fat). In addition, TCDD, PeCDD, and OCDD concentrations were also close to those observed by us (mainly in the case of dl-PCBs). The authors of this Canadian study have also shown that in some regions of Canada, the exposure to dioxins through the milk of lactating women decreased over the years 1992 to 2005.

Lu et al. (2015) have recently reported an association between a diet of women participating in their study and the concentration of POPs. According to their study, higher fish consumption translates into higher levels of dioxins in milk, resulting in a greater exposure of new-borns to PCDDs/PCDDFs and dl-PCBs. This seems to be confirmed by outcomes of our study, in which we observed a positive association between the frequency of fish consumption and the level of total WHO-TEQ_2005_ toxicity equivalent, which most probably could be attributable mainly to dioxins, as WHO-TEQ_2005_ toxicity equivalent of PCDDs/PCDFs, contrary to the WHO-TEQ_2005_ equivalent of dl-PCBs, retained such positive association with the frequency of fish consumption, once analyzed separately.

On the other hand, our outcomes seem to suggest statistically significant associations also between the frequency of consumption of dairy products and the total WHO-TEQ_2005_ equivalent as well as the WHO-TEQ_2005_ equivalent for PCDDs/PCDFs only. Nevertheless, the interpretation of these findings is peculiar as in these cases no such clear positive trend of increasing values of toxicity equivalents with increased consumption of dairy products was observed (see Table 5). Outcomes concerning such associations with the frequency of consumption of dairy products must be thus treated with caution, especially in the light of very few subjects in certain groups involved in these analyses.

## Conclusions

In this study, the concentrations of 29 compounds from the POPs group (PCDDs, PCDFs, and dl-PCBs) in the milk of lactating women living in Lodz, the third largest city in Poland, are presented. The obtained results fit well within the range of results reported by other authors throughout Europe as well as in the world. The profile of individual congeners indicates that POPs pollution occurring in the area where the research was carried out was caused mainly by traffic (highly urbanized area) or uncontrolled burning, especially garbage, unfortunately in home furnaces. This is a problem that requires constant monitoring due to the fact that the concentration of dioxins, which are very persistent, will not decrease. Their accumulation in the environment and human body will lead to progressive rise of these concentrations in years to come; thus, the exposure of new-borns drinking their mothers’ milk in the first weeks of their life could be considerably higher.

The study also reports certain interesting associations between the concentration of analyzed POPs and age as well as with the frequency of consumption of some diet components, especially fish. These associations need to be, however, treated with caution as they definitely deserve further verification in much larger studies.

## Data Availability

All datasets used and/or generated during the current study are available from the corresponding author upon reasonable request.
